# Validation of an Online Version of the Alcohol Use Disorders Identification Test (AUDIT) for Alcohol Screening in Spanish University Students

**DOI:** 10.3390/ijerph18105213

**Published:** 2021-05-14

**Authors:** Laura Ballester, Itxaso Alayo, Gemma Vilagut, José Almenara, Ana Isabel Cebrià, Enrique Echeburúa, Andrea Gabilondo, Margalida Gili, Carolina Lagares, José A. Piqueras, Miquel Roca, Victoria Soto-Sanz, Maria Jesús Blasco, Pere Castellví, Carlos G. Forero, Philippe Mortier, Jordi Alonso, on behalf of the UNIVERSAL Study Group

**Affiliations:** 1 Health Services Research Group, IMIM (Institut Hospital del Mar d’Investigacions Mèdiques), 08003 Barcelona, Spain; lballester@imim.es (L.B.); ialayo@imim.es (I.A.); gvilagut@imim.es (G.V.); mblasco@imim.es (M.J.B.); pmortier@imim.es (P.M.); 2 Department of Psychology, Girona University (UdG), 17004 Girona, Spain; 3 CIBER Epidemiología y Salud Pública (CIBERESP), 28029 Madrid, Spain; 4 Department of Biomedicine, Biotechnology and Public Health, Faculty of Nursing and Physiotherapy, University of Cádiz (UCA), 11009 Cádiz, Spain; jose.almenara@uca.es; 5 Department of Mental Health, Corporació Sanitaria Parc Taulí, 08208 Sabadell, Spain; annabel.cebria@gmail.com; 6 CIBER Salud Mental (CIBERSAM), 28029 Madrid, Spain; 7 Department of Clinical and Health Psychology, University of the Basque Country (UPV-EHU), 20018 San Sebastián, Spain; enrique.echeburua@ehu.es; 8 BioDonostia Health Research Institute, Osakidetza, 20014 San Sebastián, Spain; andrea_gabilondo@yahoo.es; 9 Institut Universitari d’Investigació en Ciències de la Salut (IUNICS-IDISBA), University of Balearic Islands (UIB), 07122 Palma de Mallorca, Spain; mgili@uib.es (M.G.); mroca@uib.es (M.R.); 10 Department of Statistic and Operational Research, Faculty of Medicine, University of Cádiz (UCA), 11009 Cádiz, Spain; carolina.lagares@uca.es; 11 Department of Health Psychology, Center for Applied Psychology, Faculty of Social and Health Sciences, Campus of Elche, Miguel Hernandez University of Elche (UMH), 03202 Elche, Spain; jpiqueras@umh.es (J.A.P.); vicky.ssanz@gmail.com (V.S.-S.); 12 School of Medicine, International University of Catalonia (UIC), 08195 Sant Cugat del Vallès, Spain; pere.castellvi.obiols@gmail.com (P.C.); cgarciaf@uic.es (C.G.F.); 13 Department of Experimental and Health Sciences, Pompeu Fabra University (UPF), 08003 Barcelona, Spain

**Keywords:** Alcohol Use Disorders Identification Test (AUDIT), university students, online survey, validity

## Abstract

Online alcohol screening may be helpful in preventing alcohol use disorders. We assessed psychometric properties of an online version of the Alcohol Use Disorders Identification Test (AUDIT) among Spanish university students. We used a longitudinal online survey (the UNIVERSAL project) of first-year students (18–24 years old) in five universities, including the AUDIT, as part of the WHO World Mental Health International College Student (WMH-ICS) initiative. A reappraisal interview was carried out with the Timeline Followback (TLFB) for alcohol consumption categories and the Mini International Neuropsychiatric Interview (MINI) for alcohol use disorder. Reliability, construct validity and diagnostic accuracy were assessed. Results: 287 students (75% women) completed the MINI, of whom 242 also completed the TLFB. AUDIT’s Cronbach’s alpha was 0.82. The confirmatory factor analysis for the one-factor solution of the AUDIT showed a good fit to the data. Significant AUDIT score differences were observed by TLFB categories and by MINI disorders. Areas under the curve (AUC) were very large for dependence (AUC = 0.96) and adequate for consumption categories (AUC > 0.7). AUDIT cut-off points of 6/8 (women/men) for moderate-risk drinking and 13 for alcohol dependence showed sensitivity/specificity of 76.2%/78.9% and 56%/97.5%, respectively. The online version of the AUDIT is useful for detecting alcohol consumption categories and alcohol dependence in Spanish university students.

## 1. Introduction

Alcohol consumption is one of the leading risk factors for disability and premature death [1,2]. Particularly, among young people, some alcohol consumption patterns are well recognized as a public health problem [3]. The last report of the European School Survey Project on Alcohol and Other Drugs (ESPAD) showed that 34% of young people reported binge drinking [4]. In Spain, according to the last report from the Survey on Alcohol and Other Drugs in Spain (EDADES) [5], the prevalence of any drinking in the last 30 days was 59.7% in young people.

Among young people, university students report a higher frequency of some risk patterns of alcohol consumption than non-students [6]. In the university population, binge drinking is common and strongly related to other risky health behaviors [7,8,9] and high levels of alcohol consumption have been reported [10,11,12,13]. In this sense, it has been proposed to carry out screening programs to identify patterns of alcohol consumption and to implement web-based interventions that have proven to be effective [14,15,16].

The Alcohol Use Disorders Identification Test (AUDIT) [17] is a self-administered instrument developed by the WHO, providing classifications of alcohol consumption and dependence. While the Spanish version of the AUDIT has been validated in health care settings [18,19,20,21], young people (12–18 years) [22,23] and university student populations [24,25], to our knowledge, this is the first study to evaluate an online version, as part of a larger mental health survey, as administered in the UNIVERSAL project [26], where the results obtained may differ according to the mode of administration [27,28]. Obtaining such evidence would be important since online instruments allow for easier and less costly administration than other modes.

The UNIVERSAL project is a multicenter, observational and prospective cohort study that aims to assess the prevalence and incidence of mental disorders among Spanish university students [26]. This project is part of the World Mental Health International College Student (WMH-ICS) initiative. The online survey administered in the study was composed of multiple screening instruments for the assessment of mental disorders and suicidal thoughts and behaviors, where the screening for alcohol use disorders (AUD) was based on the AUDIT [17,29].

The aims of the present study were to assess the reliability, construct validity and diagnostic accuracy of the online version of the AUDIT as used in the UNIVERSAL project for measuring alcohol consumption and AUD according to standard reference measures, 7-day Timeline Followback (TLFB) [30] and Mini International Neuropsychiatric Interview (MINI) [31], respectively.

## 2. Materials and Methods

### 2.1. Participants

Participants for this validation study were recruited from the UNIVERSAL project. Further information on the UNIVERSAL project has been published elsewhere [26]. Briefly, in the academic year 2014/15, all first-year students enrolled in a university degree for the first time and aged 18–24 from five Spanish universities of five Spanish regions, Andalusia (UCA), Basque Country (UPV-EHU), Balearic Islands (UIB), Catalonia (UPF) and Valencia (UMH), were eligible and they were invited to participate in the UNIVERSAL project (*n* = 16,332). These universities represented around 8% of the total number of students in public universities of Spain in the years 2014–15, and their distribution in demographic characteristics (i.e., gender, nationality and academic field) was similar to that of the overall population of students in public universities of Spain (results available upon request). The students participating in the study were re-contacted every year, from 2015/16 to 2017/18 courses, for follow-up online assessments. Ethical approval was provided by the Parc de Salut Mar—Clinical Research Ethics Committee (Reference: 2013/5252/I, 3 December 2013).

**Figure 1:**
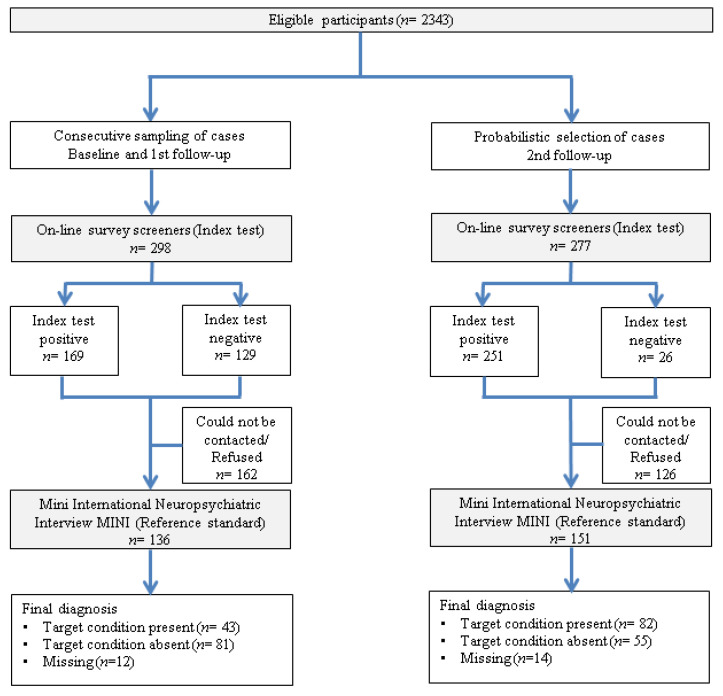
Flow diagram of the clinical reappraisal study based on STARD 2015 guidelines [33].

A total of 2343 students answered the online baseline survey of the UNIVERSAL project. In the clinical reappraisal sub-study, students were invited to participate after responding the online surveys at different time periods (i.e., at baseline and at 1st and 2nd follow-up). Inclusion criteria in the clinical reappraisal sub-study were: (i) acceptance of informed consent; (ii) provision of a telephone number; and (iii) completion of the diagnostic sections of the online survey. A sub-sample of 575 individuals fulfilling the inclusion criteria was selected for participation through sampling strategies (see flow diagram in Figure 1). Further information on the clinical reappraisal sub-study has been published elsewhere [32]. Consecutive sampling of cases was applied at baseline (2014/2015) and 1st year follow-up (2015/16). In order to increase the number of individuals with specific mental disorders for the clinical reappraisal study, a stratified random selection with different probabilities of selection was conducted during the 2nd year follow-up (2016/17) by selecting: (a) 100% of individuals who screened positive in the online survey for the following mental health problems: alcohol use disorder, generalized anxiety disorder, panic disorder, bipolar disorder, substance use disorder, suicide plan and suicide attempt; (b) a random 20% of individuals with positive screen of a major depressive episode or suicidal ideation but without any of the above mental health problems; and (c) 10% of the remaining respondents. This probabilistic sample allowed us to restore the original distribution of disorders in the UNIVERSAL study through the use of sampling weights in the analysis. The final sample of the clinical reappraisal study was 287 students.

### 2.2. Online Survey

The online survey included an adaptation of the AUDIT questionnaire [17] for estimating current usual prevalence of alcohol consumption and AUD (abuse or dependence) among first-year university students without specifying a specific time period. AUDIT is a self-administered questionnaire composed of 10 items with scoring range of 0–40 points. This questionnaire refers to the quantification of alcohol consumption, the behavior towards drinking, adverse reactions and problems related to alcohol consumption.

Three variables were defined based on the AUDIT total score: (a) binge drinking (BD), as a dichotomous variable obtained from the third AUDIT question reduced in our study to five or more drinks in both genders, “How often do you have five or more alcoholic drinks at a single sitting?”, as recommended in previous validation studies among university students [24,34]. Responses were coded as follows: never/less than once a month = 0 and 1–2 days a month/1–2 days a week/3–4 days a week/every day or nearly every day = 1 [35,36]; (b) risk drinking, where different cut-off values were established according to gender: 8 in men and 6 in women, as recommended in previous Spanish validation studies [18,21,24]; and (c) probable dependence, as a dichotomous variable from the AUDIT with a cut-off of 13 for both genders [21].

### 2.3. Reappraisal Instruments

Interviews for the clinical reappraisal study were conducted using reference standard instruments to obtain the diagnostics with which to validate the online version of the AUDIT. Telephone interviews were performed by clinical psychologists specially trained for structured interviews who were blind to the online survey responses. Interviewers had no personal information (only the telephone number) of the participants in order to preserve confidentiality. Two standardized measures were selected as gold standard instruments for this validation study: 7-day Timeline Followback (TLFB) and Mini International Neuropsychiatric Interview (MINI).

The 7-day TLFB is a drinking assessment method that obtains estimates of daily drinking in the past 7 days, using a record of standard drink units (SDUs) consumed at different times or occasions throughout the day [30]. Participants completed a diary, with the help of the clinical interviewer, in which they were asked about the amount of alcohol consumed at different times of the day during the previous seven days. The following four categories were considered: (a) non-drinkers (SDUs = 0), (b) low-risk drinkers (SDUs ≤ 21 and ≤14 for men and women, respectively) [37], (c) moderate-risk drinkers (22–27 for men and 15–16 SDUs for women) and (d) high-risk drinkers (≥28 and 17 SDUs) [37,38]. Additionally, BD was defined as the consumption of 5 or more SDUs in a single sitting [38,39,40].

The adapted version of the Spanish structured interview MINI 5.0.0 [41] was administered for AUD diagnostics according to the Diagnostic and Statistical Manual of Mental Disorders (DSM-IV) [42] criteria and referred to the previous 12 months. Four categories were considered: (a) non-case; (b) alcohol abuse; (c) alcohol dependence; and (d) any AUD (abuse/dependence).

### 2.4. Analysis

We compared characteristics of the clinical reappraisal sub-sample and prevalence estimates of alcohol consumption categories and AUD according to the reference standards by gender using a chi-squared test and Fisher’s exact test. Reliability and confirmatory factor analysis was performed in the overall sample of participants in the UNIVERSAL project (*n* = 2343). The analysis with MINI as a reference standard was performed with the whole sample of reappraised university students (*n* = 287) and the analysis with TLFB as a reference standard was restricted to those students from the reappraisal sample who provided all TFLB data (*n* = 242).

The reliability of AUDIT was analyzed using Cronbach’s alpha and Guttman’s lambda-2, as measures of internal consistency. The AUDIT total score was used in this study and its unidimensionality was evaluated through confirmatory factor analysis (CFA) with the one-factor solution, using unweighted least squares estimation. In addition to the chi-square statistic, which is sensitive to sample size [43,44], we assessed the chi-square statistic and degrees of freedom and its corresponding *p*-value. Given the sensitivity of this test to large sample sizes, we additionally examined the following goodness of fit indices: comparative fit index (CFI), Tucker–Lewis index (TLI) and root mean square error of approximation (RMSEA), considering the cut-off criteria of 0.95 in CFI and TLI for good fit and RMSEA < 0.06 for good fit [45,46].

Known-groups validity was assessed by computing weighted average scores (weighted standard deviation) of AUDIT across TLFB groups: those who do not drink; low-risk drinkers and moderate-risk to high-risk drinkers. Similarly, we computed weighted average scores (weighted standard deviation) of AUDIT across MINI diagnosis: no AUD, alcohol abuse and alcohol dependence disorder. A Jonckheere–Tepstra test was calculated with the ex ante hypothesis that there would be a gradient from lower to higher values in AUDIT scores across these groups. Statistical significance was set at the 5% level based on two-sided tests. Cohen’s effect sizes were computed for each category as compared to the lowest category (“non-drinkers” for TLFB; “non-case” for MINI) [47] considering small (0.2), moderate (0.5) and large (0.8) effect sizes [48]. Criterion validity of the AUDIT scores was assessed with the receiver operating characteristic (ROC) and its corresponding area under the curve (AUC), considering the TLFB definitions and MINI diagnoses as the reference standards. According to Landis and Koch (1977), different ranges of AUC were assigned labels of discrimination ability: slight (0.50–0.59), fair (0.6–0.69), moderate (0.7–0.79), substantial (0.8–0.89) and almost perfect (≥0.9) [49]. Finally, we studied test characteristics for pre-specified cut-off points of the AUDIT with respect to TLFB and MINI definitions described previously: sensitivity (SN), specificity (SP), positive predictive value (PPV), negative predictive value (NPV), likelihood ratio positive (LR+) and likelihood ratio negative (LR-). The AUCs for the dichotomous categories of the AUDIT are also presented. In the case of a dichotomous predictor and a dichotomous outcome, the AUC equals (SN + SP)/2 [50]. For assessing the differences in the prevalence between the online version of the AUDIT and reference standard, a McNemar χ^2^ test was calculated.

Inverse probability weighting was computed to adjust the sampling method applied in the reappraisal selection carried out during the 2nd year follow-up (2016/17). Weights were obtained as the inverse of the probability of selection within each stratum in 2nd year follow-up and normalized to the total sample size of the clinical reappraisal study. Post-stratification weights were applied for the correction of differences of gender, academic field and nationality characteristics between the clinical reappraisal sample and the respective UNIVERSAL sample, as the reference population. Analyses were performed using SAS v9.4 [51] and MPLUS v8.5 [52].

## 3. Results

Table 1 shows the sociodemographic characteristics and prevalence of the reference standard measures in the clinical reappraisal sample. The majority of the sample was women (75.3%), 69.6% were 18 years old and 2.9% had non-Spanish nationality. Most students (47.6%) were enrolled in social sciences. According to the MINI, men were significantly more likely than women to meet the criteria of alcohol abuse (14.2% vs. 6.4%, *p* = 0.028) but no gender differences for MINI alcohol dependence were found (1.6% vs. 1.3%). TLFB alcohol consumption categories (i.e., binge drinking, moderate-risk drinking and high-risk drinking) also did not show statistically significant differences between genders.

The internal consistency of AUDIT evaluated by the Cronbach’s alpha coefficient was 0.817. The lambda-2 coefficient was 0.829. Corrected item-total correlations ranged from 0.332 to 0.663 (Table 2).

Table 3 shows standardized factor loadings of the one-factor CFA model of the online version of the AUDIT, which ranged from 0.545 to 0.797. The model chi-square statistic was 219.073 (35), *p*-value < 0.001, and the CFA indices had optimal values according to the cut-off criteria, indicating a good fit to the data, with a CFI of 0.973, a TLI of 0.966 and an RSMEA of 0.049 (95%CI 0.043–0.056).

**Figure 2:**
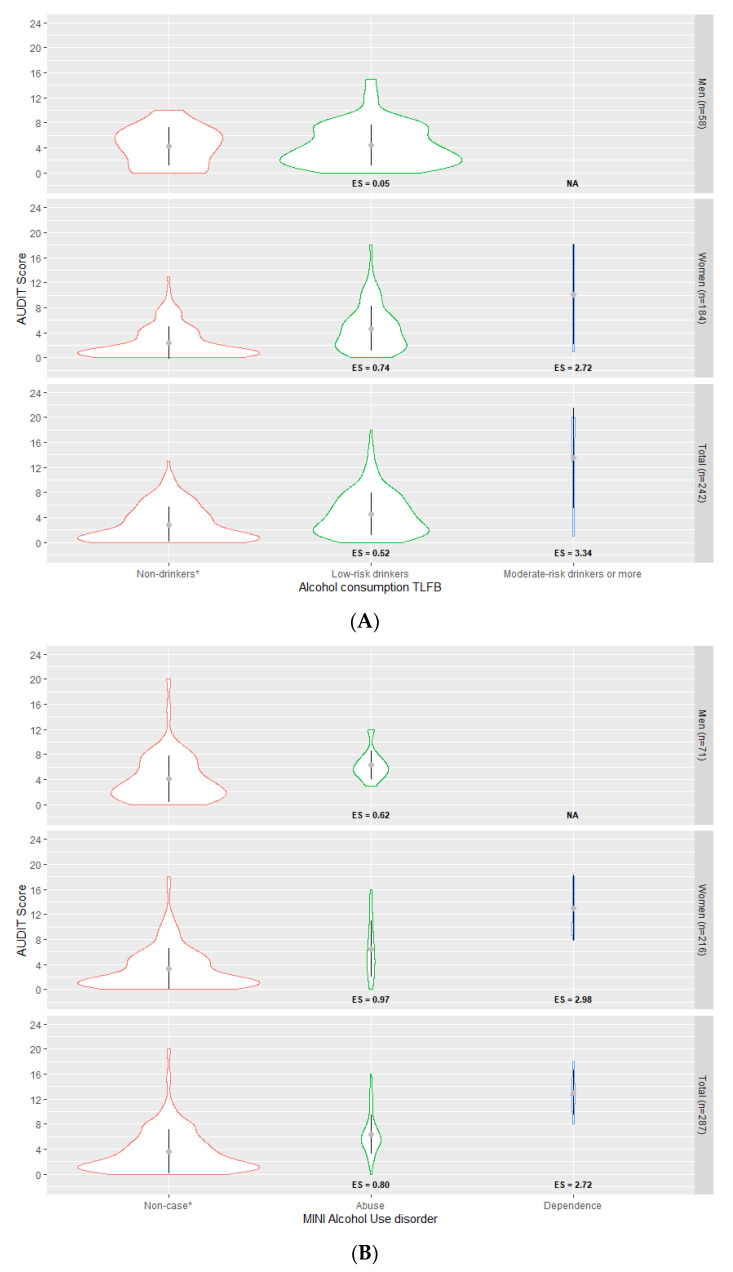
(**A**) AUDIT online mean scores (standard deviation) and effect size, by alcohol consumption categories (TLFB) (*n* = 242). Weighted values. AUDIT: Alcohol Use Disorders Identification Test; TLFB: Timeline Followback; ES: effect size. * Reference category. Jonckheere–Terpstra test = 10,025.5; *p*-value < 0.001. ANOVA with Tukey post hoc test *p*-value < 0.001. (**B**) AUDIT online mean scores (standard deviation) and effect size, by alcohol use disorder (MINI) (*n* = 287). Weighted values. AUDIT: Alcohol Use Disorders Identification Test; MINI: Mini International Neuropsychiatric Interview; ES: effect size. * Reference category. Jonckheere–Terpstra test = 6337.5; *p*-value < 0.001. ANOVA with Tukey post hoc test *p*-value < 0.001.

Figure 2A shows weighted mean AUDIT scores and their weighted standard deviation (SD), as well as corresponding effect sizes, across the TLFB alcohol consumption categories. A clear upward gradient was observed for the AUDIT scores, rising from the “non-drinkers” group (mean = 2.86, SD = 2.89), through to the “low-risk drinkers” (mean = 4.51, SD = 3.42) and finally the “moderate-risk drinkers or more” group (mean = 13.5, SD = 8.03) (J = 10,025.5; *p*-value < 0.001). Similar results were obtained for women. Results for men could not be calculated due to insufficient data in the “moderate-risk drinkers or more” category (*n* < 5), but differences between non-drinkers and low-risk drinkers were small and not statistically significant. Effect sizes associated with “moderate-risk drinkers or more” were the highest for the total sample (ES = 3.34) and for women (ES = 2.72). Figure 2B shows weighted mean AUDIT scores and weighted SD for alcohol abuse and dependence as assessed by the MINI. Again, a consistent upward gradient was observed for the AUDIT scores, rising from “non-cases” to respondents with “dependence” criteria. Results for men in the “dependence” category (*n* < 5) could not be calculated due to insufficient data. Effect sizes associated with “dependence” were the highest for the total sample (ES = 2.72) and for women (ES = 2.98).

**Figure 3:**
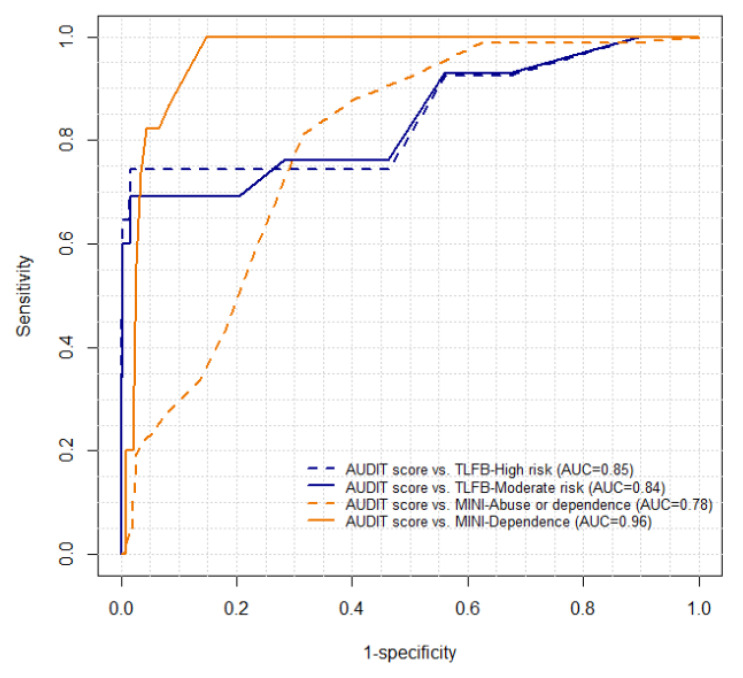
ROC curves and AUC between continuous scores of AUDIT and MINI/TLFB (*n* = 287). AUDIT: Alcohol Use Disorders Identification Test; TLFB: Timeline Followback; MINI: Mini International Neuropsychiatric Interview; ROC: receiver operating curve; AUC: area under the ROC curve.

The ability of the AUDIT scores for detecting alcohol risk-drinking and AUD, using TLFB and the MINI as the respective gold standards, is presented by ROC curves and AUCs in Figure 3. AUCs were substantial for the TLFB, with values of 0.84 to detect moderate-risk drinking and 0.85 for high-risk drinking. For the MINI, AUCs ranged from fair (0.78) for alcohol abuse/dependence to almost perfect (0.96) for alcohol dependence.

Accuracy analyses were first performed for alcohol consumption categories (Table 4), comparing different AUDIT cut-off points with the Timeline Followback categories as the gold standard. AUDIT cut-off points had a sensitivity (SN) of 41.4% and specificity (SP) of 83.6% for detecting binge drinking. Results for pre-specified cut-off points for detecting at least moderate-risk drinkers were SN = 76.2% and SP = 78.9% for cut-off point 8 for men and cut-off point 6 for women. Using the cut-off point 13 for both genders provided an SN of 74.4% and SP of 98.3% for high-risk drinkers based on the TLFB. Prevalence estimates are also presented in Table 4, which show statistically significant differences between the index text and gold standard according to the McNemar test for binge drinking and for moderate-risk drinkers. The AUCs were fair to substantial for moderate-risk and high-risk drinking (ranging from 0.7 to 0.9), and fair for binge drinking (0.6).

In Table 5, AUDIT cut-off points were compared to the MINI as the gold standard for detecting AUD. The AUDIT cut-off point used for detecting alcohol abuse or dependence was 8, as a generally accepted cut-off [29,53]. SN for men was 26.6% and for women 46%, while SP was higher for both genders (81.1%; 90.2%, respectively). The alternative cut-off point of 13 for alcohol dependence, recommended by García-Carretero et al. (2016), showed more accurate results: SN for the overall sample 56% and 54.3% for women, and SP of 97.5% and 97.6%, respectively [24]. According to the McNemar test (Table 5), no statistically significant differences were found in prevalence estimates. The dichotomous AUCs for alcohol dependence were slightly higher than values for alcohol abuse/dependence for the overall sample (0.60 vs. 0.77) and women (0.68 vs. 0.76).

## 4. Discussion

This study has assessed the validity of the online AUDIT to identify diagnostic criteria for AUD as well as risk-drinking according to the MINI and the TLFB. The results show that the online version of the AUDIT is adequate to detect alcohol dependence among Spanish university students and to discriminate different alcohol consumption categories.

Reliability is a prerequisite for validity [54]. Internal consistency for the online version of the AUDIT was good on the whole measurement, which reflected the consistency of responses across the items of the instrument. Our results are comparable to those found in previous Spanish studies among university students [24] and the general population [18,19], which found a value of around 0.8. However, corrected item-total correlations were low for some of the items, particularly for items 6 and 10, which were also found in previous studies [19,24] that concluded that this could be because the 4th to 10th items assess dependence and harmful alcohol use [29] and these were less frequent in this population.

The unidimensionality evaluation, consistent with the total score of the AUDIT, showed a good fit of the results, as found in previous studies [55,56]. The results obtained for the known-groups comparisons also provide support for the construct validity of the online version of the AUDIT. An upward gradient was observed in both cases, for alcohol consumption and for AUD categories. Increasing scores were obtained across different types of consumption (similar to those reported by García-Carretero et al., 2016).

The results reported also offer evidence of good diagnostic accuracy of the AUDIT for identifying risk-drinking categories with the TLFB and alcohol dependence (assessed by the MINI). The AUCs to assess discrimination ability for risk-drinking were substantial (0.84–0.86) and similar to those found in previous validation studies among university students (AUCs 0.87–0.93) [34,57]. However, these AUCs to identify risk-drinking were slightly lower than those obtained in previous Spanish validation studies (AUC = 0.95–0.98) [20,24], which differed in the mode of administration of the AUDIT. The AUC for detecting alcohol dependence was almost perfect (AUC = 0.96), similar to AUC values obtained in other studies [24,57]. Finally, the AUC for detecting BD with the AUDIT was fair (0.6) and lower than the AUC, which was found by Cortés et al. (2017), who support the recommendation to change the third item of the AUDIT to four or more drinks in women [34] or using the full instrument to identify BD, such as AUDIT or AUDIT-C [23,25].

In this study, previously suggested cut-off points in AUDIT for alcohol risk consumption among men and women (8 and 6, respectively) resulted in sensitivities and specificities lower than the Spanish validation among university students [24] and primary care [18,21], but similar to the validation carried out in the United States among university students by Kokotailo et al. (2004) [34]. The online AUDIT cut-off score of 13 for detecting alcohol dependence also showed lower psychometric properties than another previous Spanish validation study [24]. Sensitivity analyses conducted in this study showed better psychometric properties with a cut-off point of 12 for detecting alcohol dependence (full results of the additional analyses are available upon request). Additionally, our study showed low PPVs and NPVs, which might be due to the low prevalence of alcohol disorders in our population [58].

The results of this study must be interpreted taking into account the following limitations. First, we used the MINI as the gold standard diagnostic instrument, which is not used as widely as other structured interviews (such as the Structured Clinical Interview DSM-IV (SCID) [59] or Composite International Diagnostic Interview (CIDI) [60]), but we applied it for feasibility and because it has shown acceptable SN/SP values (0.8/0.8) for structured interviews. Second, also in relation to the MINI, this study used the validated Spanish version of the MINI which is based on the DSM-IV criteria. Due to the subsequent publication of the DSM-5 and the changes in the diagnosis of alcohol use disorder, the results obtained in this study may be different from the new criteria. Recent validation studies of the AUDIT according to the DSM-5 have found few differences in their results [61,62,63,64], but further research on the validation of the AUDIT among university students is needed. Third, although the validity of the online instrument was established in a sample of 287 university students, the low prevalence of alcohol-related problems limited the statistical power of our study. Importantly, our results on the overall sample are consistent with previous findings [24]. However, studies with larger samples are needed for the online version of the AUDIT. A limitation concerns the recall periods of the instruments. The MINI interview administered in this study uses a 12-month recall period and the TLFB that was administered in this study uses a recall period of 1 week. On the other hand, we used an adapted version of the AUDIT without the original 12-month recall period, thus assessing current usual alcohol consumption without a specific reference period. We decided to use a short recall period of the TLFB to reduce the possible bias in the information collected regarding long periods of time and to reduce the burden on the interviewer and the respondent of a longer diary [65,66,67,68]. However, the use of such a short period of time could bias the estimate of the usual consumption pattern of university students [69]. If this was the case, the association among the measures would be attenuated and would underestimate the validity of the online AUDIT.

## 5. Conclusions

We have tested the metric performance of the Spanish online version of the AUDIT among Spanish university students. The results indicate good reliability of this version, as well as good construct validity and diagnostic accuracy. If applied in epidemiological research settings, the online version of the AUDIT might be useful to improve the detection of risk alcohol consumption patterns and probable cases of AUD diagnosis. The ease of administering the online AUDIT will facilitate its inclusion in more complete mental health profile evaluations. However, there is a shorter validated version of the AUDIT (i.e., the AUDIT-C) that could be useful, so a next step would be to validate the online version of the AUDIT-C among university students. It is known that online screening and interventions could reduce drinking in university students [70]. Such programs could be implemented more widely, for instance, among university campuses.

## Figures and Tables

**Table 1:** Characteristics of the clinical reappraisal sub-sample and prevalence estimates ^ of alcohol consumption and alcohol use disorder according to the TLFB and the MINI, by gender. (Unweighted observations and weighted percentages.)

	Total *n* = 287	Men *n* = 71	Women *n* = 216	*p*-Value
	*n* (%)	*n* (%)	*n* (%)	
Age				
18	177 (69.6)	45 (76.0)	132 (64.4)	0.09
19	54 (14.5)	13 (11.6)	41 (16.9)	
20+	56 (15.9)	13 (12.4)	43 (18.8)	
Nationality				
Non-Spanish	29 (2.9)	4 (2.3)	25 (3.2)	0.74
Field of studies				
Arts and Humanities	37 (9.8)	2 (7.0)	35 (11.9)	<0.01 *
Engineering and Architecture	32 (18.6)	21 (32.0)	11 (8.2)	
Health Sciences	85 (15.7)	14 (10.2)	71 (20.1)	
Science	25 (8.4)	11 (8.6)	14 (8.2)	
Social and Legal Sciences	108 (47.6)	23 (42.2)	85 (51.6)	
AUDIT categories				
Binge drinking ^a^	45 (18.9)	14 (25.4)	31 (13.6)	0.02 *
Risk drinking ^b^	65 (21.9)	12 (20.2)	53 (23.3)	0.53
Probable dependence ^c^	11 (3.3)	3 (3.5)	8 (3.2)	0.76
TLFB Alcohol consumption				
Binge drinking ^d^	32 (9.8)	8 (9.6)	24 (10.0)	0.93
Low-risk drinkers ^e^	112 (51.4)	34 (65.9)	78 (39.6)	<0.01 *
Moderate-risk drinkers or more ^f^	7 (2.0)	1 (1.8)	6 (2.1)	1.0
High-risk drinkers ^g^	6 (2.0)	1 (1.8)	5 (2.1)	1.0
MINI alcohol use disorder				
Abuse ^h^	29 (10.0)	11 (14.2)	18 (6.4)	0.03 *
Dependence ^i^	7 (1.5)	2 (1.6)	5 (1.3)	1.0
Abuse/Dependence ^j^	35 (11.2)	13 (15.6)	22 (7.6)	0.03 *

TLFB: Timeline Followback; MINI: Mini International Neuropsychiatric Interview. ^a^ Third question of the AUDIT. ^b^ AUDIT cut-off point 6/8 (females/males). ^c^ AUDIT cut-off point 13. ^d^ TLFB: ≥5 SDU/per occasion. ^e^ TLFB: <22 SDU males and <15 SDU females. ^f^ TLFB: ≥22 SDU/males and ≥15 SDU/females. ^g^ TLFB: ≥28 SDU/males and ≥17 SDU/females. ^h^ MINI alcohol abuse. ^i^ MINI alcohol dependence. ^j^ MINI alcohol abuse or dependence. ^ Prevalence estimates according to the reappraisal temporary moment (see the Materials and Methods section). *p*-values obtained from chi-squared test and Fisher’s exact test when cells are *n* < 5. * *p*-value statistically significant at 0.05.

**Table 2:** Internal reliability of AUDIT online questionnaire administered in the UNIVERSAL sample (*n* = 2343).

AUDIT Items	Mean (SD)	Corrected Item-Total Correlation	Cronbach’s Alpha If Item Deleted
1	1.69 (0.67)	0.539	0.798
2	0.61 (0.76)	0.509	0.800
3	0.78 (0.88)	0.663	0.781
4	0.29 (0.77)	0.520	0.798
5	0.27 (0.53)	0.558	0.796
6	0.18 (0.53)	0.332	0.816
7	0.46 (0.69)	0.502	0.800
8	0.41 (0.66)	0.623	0.786
9	0.27 (0.79)	0.446	0.808
10	0.06 (0.35)	0.339	0.816

AUDIT: Alcohol Use Disorders Identification Test; SD: standard deviation. Cronbach’s alpha: 0.817. Guttman’s lambda-2: 0.829.

**Table 3:** Standardized factor loadings from a confirmatory factor analysis with one factor of the online version of the AUDIT administered in the UNIVERSAL sample (*n* = 2343).

AUDIT Items	Standardized Factor Loadings	
	Estimate	SE
1	0.696	0.021
2	0.622	0.020
3	0.777	0.015
4	0.759	0.019
5	0.753	0.016
6	0.545	0.026
7	0.688	0.019
8	0.797	0.014
9	0.659	0.023
10	0.569	0.032
RMSEA (95% CI)	0.049 (0.043–0.056)	
CFI	0.973	
TLI	0.966	
χ^2^ (DF), *p*-value	219.073 (35), *p* < 0.001	

AUDIT: Alcohol Use Disorders Identification Test; RMSEA: root mean square error of approximation; CFI: comparative fit index; TLI: Tucker–Lewis index; χ^2^: chi-square statistic; CI: confidence interval; DF: degrees of freedom.

**Table 4:** Operating characteristics of AUDIT online questionnaire cut-off points for estimating reference standard (TLFB) prevalence of alcohol consumption in standard drink units (SDUs) per week by gender (weighted values).

		AUDIT	TLFB	Positive Operating Characteristics			Negative Operating Characteristics			McNemar	AUC
	Cut-Off Point	% (SE)	% (SE)	SN (SE)	PPV (SE)	LR+	SP (SE)	NPV (SE)	LR−	χ^2^ (*p*-Value)	
Total (*n* = 242)											
Binge drinking ^a^		18.9 (2.5)	9.8 (1.9)	41.4 (9.9)	21.6 (5.9)	2.5	83.6 (2.4)	92.9 (1.8)	0.7	10.1 (0.002 *)	0.63
Moderate-risk drinkers or more ^b^	6/8	22.2 (2.7)	2 (0.9)	76.2 (19.0)	6.9 (3.4)	3.6	78.9 (2.6)	99.4 (0.6)	0.3	48.91 (<0.001 *)	0.78
High-risk drinkers ^c^	13	3 (1.1)	1.9 (0.9)	74.4 (19.5)	46.2 (17.6)	43.8	98.3 (0.8)	99.5 (0.4)	0.3	1.58 (0.209)	0.86
Men (*n* = 58) ^											
Binge drinking ^a^		25.4 (5.7)	9.5 (3.9)	50.7 (15.8)	19.0 (7.4)	2.2	77.3 (4.1)	93.7 (2.6)	0.6	11.3 (0.001 *)	0.64
Women (*n* = 184)											
Binge drinking ^a^		13.6 (2.5)	10.1 (2.2)	34.4 (12.7)	25.5 (10.0)	3	88.7 (2.8)	92.4 (2.4)	0.7	1.05 (0.30 *)	0.62
Moderate-risk drinkers or more ^d^	6	23.2 (3.1)	2.4 (1.1)	63.9 (27.7)	6.6 (4.3)	2.9	77.8 (3.5)	98.9 (1.0)	0.5	27.06 (<0.001 *)	0.71
High-risk drinkers ^e^	13	3 (1.3)	2.1 (1.1)	59.6 (28.3)	43.0 (24.8)	35.1	98.3 (1.1)	99.1 (0.8)	0.4	0.37 (0.542)	0.79

AUDIT: Alcohol Use Disorders Identification Test; TLFB: Timeline Followback; SN: sensitivity; PPV: positive predictive value; LR+: likelihood ratio positive; SP: specificity; NPV: negative predictive value; LR−: likelihood ratio negative; SE: standard error; AUC: area under the receiver operating characteristic curve. ^a^ Third question in AUDIT online; TLFB: ≥5 SDU/per occasion; ^b^ TLFB: ≥22 SDU males and ≥15 SDU females; ^c^ TLFB: ≥28 SDU males and ≥17 SDU females; ^d^ TLFB: ≥15 SDU; ^e^ TLFB: ≥17 SDU. ^ No sufficient data (*n* < 5) to calculate “moderate-risk drinkers” and “high-risk drinkers”. * *p*-value statistically significant at 0.05.

**Table 5:** Operating characteristics of AUDIT online questionnaire cut-off points for estimating reference standard (MINI) prevalence of alcohol dependence and alcohol abuse or dependence by gender (weighted values).

		AUDIT	MINI	Positive Operating Characteristics			Negative Operating Characteristics			McNemar	AUC
	Cut-Off Point	% (SE)	% (SE)	SN (SE)	PPV (SE)	LR+	SP (SE)	NPV (SE)	LR−	χ^2^ (*p*-Value)	
Total (*n* = 287)											
Alcohol abuse/dependence ^a^	8	15.9 (2.2)	11.2 (1.9)	33.9 (8.4)	23.9 (6.3)	2.5	86.3 (2.2)	91.2 (1.8)	0.8	3.23 (0.072)	0.60
Alcohol dependence ^b^	13	3.3 (1.1)	1.5 (0.7)	56 (24.8)	24.9 (14.4)	22.4	97.5 (0.9)	99.3 (0.5)	0.5	3.12 (0.077)	0.77
Men (*n* = 71) ^											
Alcohol abuse/dependence ^a^	8	20.1 (4.8)	15.6 (4.3)	26.6 (9.9)	20.7 (8.1)	1.4	81.1 (3.8)	85.7 (3.5)	0.9	0.93 (0.334)	0.54
Women (*n* = 216)											
Alcohol abuse/dependence ^a^	8	12.6 (2.3)	7.7 (1.8)	46 (13.8)	28.1 (10.0)	4.7	90.2 (2.5)	95.3 (1.8)	0.6	2.86 (0.091)	0.68
Alcohol dependence ^b^	13	3.2 (1.2)	1.5 (0.8)	54.3 (35.2)	26.4 (19.7)	22.6	97.6 (1.2)	99.3 (0.7)	0.5	1.39 (0.239)	0.76

AUDIT: Alcohol Use Disorders Identification Test; MINI: Mini International Neuropsychiatric Interview; SN: sensitivity; PPV: positive predictive value; LR+: likelihood ratio positive; SP: specificity; NPV: negative predictive value; LR−: likelihood ratio negative; SE: standard error; AUC: area under the receiver operating characteristic curve. ^a^ MINI alcohol abuse or dependence. ^b^ MINI alcohol dependence. ^ No sufficient data (*n* < 5) to calculate “alcohol dependence”.

## Data Availability

The de-identified dataset containing the necessary variables to reproduce all numbers reported in the article will be deposited in an external online repository upon acceptance of the article for publication.
